# Serum Amyloid A Receptor Blockade and Incorporation into High-Density Lipoprotein Modulates Its Pro-Inflammatory and Pro-Thrombotic Activities on Vascular Endothelial Cells

**DOI:** 10.3390/ijms160511101

**Published:** 2015-05-15

**Authors:** Belal Chami, Nicola Barrie, Xiaoping Cai, Xiaosuo Wang, Moumita Paul, Rebecca Morton-Chandra, Alexandra Sharland, Joanne M. Dennis, Saul B. Freedman, Paul K. Witting

**Affiliations:** 1 Discipline of Pathology, Sydney Medical School, the University of Sydney, Sydney, NSW 2006, Australia; E-Mails: belal.chami@sydney.edu.au (B.C.); nicola.gbarrie@gmail.com (N.B.); xcai4874@uni.sydney.edu.au (X.C.); xiaosuo.wang@sydney.edu.au (X.W.); martindennis@optusnet.com.au (J.M.D.); 2 Transplantation Immunobiology Group, Central Clinical School, Sydney Medical School, the University of Sydney, Sydney, NSW 2006, Australia; E-Mails: moumita.paul@sydney.edu.au (M.P.); becm79@hotmail.com (R.M.-C.); alexandra.sharland@sydney.edu.au (A.S.); 3 Sydney Medical School, the University of Sydney, ANZAC Research Institute, Concord Repatriation General Hospital, Sydney, NSW 2139, Australia; E-Mail: ben.freedman@sydney.edu.au

**Keywords:** serum amyloid A, inflammation, atherosclerosis, high-density lipoprotein

## Abstract

The acute phase protein serum amyloid A (SAA), a marker of inflammation, induces expression of pro-inflammatory and pro-thrombotic mediators including ICAM-1, VCAM-1, IL-6, IL-8, MCP-1 and tissue factor (TF) in both monocytes/macrophages and endothelial cells, and induces endothelial dysfunction—a precursor to atherosclerosis. In this study, we determined the effect of pharmacological inhibition of known SAA receptors on pro-inflammatory and pro-thrombotic activities of SAA in human carotid artery endothelial cells (HCtAEC). HCtAEC were pre-treated with inhibitors of formyl peptide receptor-like-1 (FPRL-1), WRW4; receptor for advanced glycation-endproducts (RAGE), (endogenous secretory RAGE; esRAGE) and toll-like receptors-2/4 (TLR2/4) (OxPapC), before stimulation by added SAA. Inhibitor activity was also compared to high-density lipoprotein (HDL), a known inhibitor of SAA-induced effects on endothelial cells. SAA significantly increased gene expression of TF, NFκB and TNF and protein levels of TF and VEGF in HCtAEC. These effects were inhibited to variable extents by WRW4, esRAGE and OxPapC either alone or in combination, suggesting involvement of endothelial cell SAA receptors in pro-atherogenic gene expression. In contrast, HDL consistently showed the greatest inhibitory action, and often abrogated SAA-mediated responses. Increasing HDL levels relative to circulating free SAA may prevent SAA-mediated endothelial dysfunction and ameliorate atherogenesis.

## 1. Introduction

The pathogenesis of atherosclerosis encompasses arterial wall inflammation, accumulation of native and oxidised lipids, plaque formation and thrombosis [1]. Although atherogenesis may take decades to manifest as symptomatic cardiovascular disease (CVD), the earliest phase involves vascular endothelial cell dysfunction [2,3].

Arterial endothelial dysfunction leads to loss of barrier function that promotes inflammatory cell uptake and lipid accumulation. The exact cause of endothelial dysfunction leading to loss of barrier function is not known but may be linked to upregulation of cellular adhesion molecules [4,5,6], altered production and bioactivity of endothelium-derived nitric oxide (NO) [7] and accumulation of reactive oxygen species through a mechanism of unregulated production and decreased ability to neutralise damaging oxidants [8]. Pro-inflammatory and pro-thrombotic stimuli also adversely affect endothelial function. For example, the inflammatory cytokine tumour necrosis factor (TNF) promotes the expression of adhesion molecules on endothelial cells and induces tissue factor (TF), itself linked to the development of atherosclerotic plaque and subsequent thrombus formation [9,10,11].

The acute phase protein, serum amyloid A (SAA), is markedly upregulated (up to 1000-fold) in response to infection and during chronic inflammation [12,13,14,15] and predicts adverse events in patients with vascular disease. SAA is also found within thrombus material and at sites of ruptured plaques [16]. SAA can stimulate vascular cells to express cytokines, chemokines, adhesion molecules and matrix metalloproteinases [17,18,19], which are linked to the development of atherosclerosis. Recent studies have implicated a causal role of SAA as a pro-inflammatory and pro-thrombotic mediator in the pathogenesis of atherosclerosis [20,21,22,23]. We [24], and others [25] have shown that SAA’s potent pro-atherogenic affects on the endothelium include the induction of the transcription factor, nuclear factor κ B (NFκB), which is implicated in the regulation of pro-inflammatory and pro-thrombotic stimuli. Cytokines and chemokines induced by SAA are linked to an increased production of superoxide radical anion by endothelial cells that impairs NO bioactivity and endothelial function [24,25].

The importance of SAA in several acute, pathological and chronic conditions, has led to investigations aimed at elucidating the mechanism of SAA’s interactions in target cells. To date, several proteins have been identified as receptors that may mediate SAA binding and internalisation in vascular cells. The G-coupled formyl peptide receptor like-1 (FPRL-1) has been demonstrated to mediate SAA-induced chemotaxis and cytokine release in neutrophils [26], while toll-like receptors (TLRs) 2/4 have been identified as novel SAA receptors mediating activities such as pro-inflammatory cytokine expression in macrophages (TLR2, [27]) and NO production via MAPK/ERK signalling pathways in macrophages (TLR4, [28]). SAA also appears to be a ligand for the receptor for advanced glycation end products (RAGE) [29].

The activities of SAA may be affected by its binding to high-density lipoprotein (HDL) [24,30] although not all proposed regulators of SAA activity bind the acute phase protein or compete with SAA receptor activation [31]. Circulating SAA is normally found as an apolipoprotein in HDL [32]. Interactions between SAA and HDL are complex and may impact on the biological activity of these individual components. For example, HDL attenuates the pro-inflammatory and pro-thrombotic actions of SAA in endothelial cells [22,24]. Conversely, SAA may adversely affect the anti-atherogenic qualities of HDL. Thus, SAA displaces apolipoproteins in HDL, including the major apolipoprotein ApoA-I [33], affecting HDL participation in lipid transport and metabolism and promoting pro-atherogenic proteoglycan binding to the vascular wall [34]. SAA enrichment of HDL may also reduce the anti-inflammatory properties of HDL [35], as released ApoA-I may decrease arterial inflammation [36].

The development of subclinical atherosclerosis and endothelial dysfunction in human carotid arteries may be linked to the progression of CVD. For example, the extent of intima-to-media thickening of the carotid artery may be a predictor of stroke [37], whereas the extent of carotid plaque formation (assessed by plaque score) rather than carotid intima-to-media thickness is a better predictor for coronary artery disease [38]. Due to the atherogenic potential of SAA-mediated signalling on the vascular endothelium, we examined the effectiveness of inhibiting SAA activity in human carotid artery endothelial cells (HCtAEC) with various pharmacological inhibitors targeting FPRL-1, RAGE and TLR2/4. We also compared pharmacological receptor inhibition with the action of freshly isolated HDL, which binds SAA and subsequently quenches SAA activity.

## 2. Results

### 2.1. SAA Receptor Inhibitor/Antagonists and HDL Suppress SAA-Induced Pro-Atherogenic Gene Expression in Endothelial Cells

**Figure 1:**
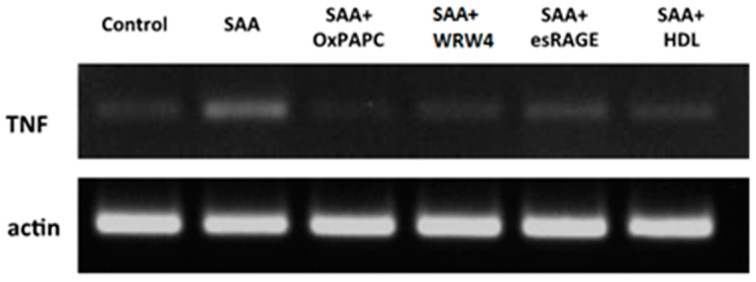
Suppression of SAA-induced TNF gene expression by pharmacological agents and HDL. Cultured HCtAEC were treated with either HBSS alone (control) or pre-incubated with the indicated pharmacological inhibitor (WRW4, 30 µg/mL; esRAGE, 15 µg/mL and OxPap C, 25 or 45 µg/mL) prior to the addition of SAA (10 μg/mL). Cells were then incubated at 37 °C and after 4.5 h the cells assessed for expression of TNF and β-Actin (house keeping gene). Gel images are representative of *n =* 6 individual experiments.

SAA induces the expression of pro-inflammatory and pro-thrombotic factors in peripheral blood mononuclear cells [22] and endothelial cells [24,39]. Consistent with these data, mRNA levels of pro-inflammatory genes, TF and TNF were significantly increased (*p <* 0.001; ~4.5-fold, and ~7-fold, respectively) following treatment of cultured HCtAEC’s cells with SAA (Figure 1 and Table 1). NFκB gene expression was also increased in HCtAECs after SAA treatment (*p <* 0.001) indicating that SAA may mediate TF and TNF gene expression via activation of NFκB [39].

**Table 1:** HDL suppresses SAA-induced pro-inflammatory and pro-thrombotic gene expression in HCtAEC ^a^.

Treatment								
Gene	Control	SAA	OxPap C (25 μg/mL)	OxPap C (45 μg/mL)	WRW4	OxPap C + WRW4	esRAGE	HDL
**TF**	1 ± 0.02	4.4 ± 0.6 *	3.4 ± 0.3 *^,#^	3.2 ± 0.1 *^,#^	3.9 ± 0.1 *	3.3 ± 0.5 *^,#^	3.1 ± 0.2 *^,#^	0.9 ± 0.4 ^#^
**NFκB**	1 ± 0.02	3.4 ± 0.3 *	2.2 ± 0.3 *^,#^	2.0 ± 0.3 *^,#^	2.4 ± 0.4 *^,#^	1.9 ± 0.2 *^,#^	3.3 ± 0.2 *	1.4 ± 0.4 ^#^
**TNF-α**	1 ± 0.17	6.8 ± 0.6 *	2.7 ± 0.4 *^,#^	2.6 ± 0.9 *^,#^	3.7 ± 0.7 *^,#^	2.3 ± 0.3 *^,#^	5.5 ± 1.2 *	1.7 ± 0.3 ^#^

^a^ Cultured HCtAEC were treated with vehicle (control) or pre-incubated with a pharmacological inhibitor (OxPap C; 25 μg/mL, OxPap C; 45 μg/mL, WRW4; 30 μg/mL, OxPap C + WRW4; 25 μg/mL OxPap C + 30 μg/mL WRW4, esRAGE; 25 μg/mL, freshly isolated HDL; 250 μg protein/mL) prior to the addition of SAA (10 μg/mL). Relative mRNA expression levels were standardised against corresponding β-Actin and expressed as a fold-change relative to control. Data was quantified using densitometry and represents mean ± SD of *n =* 3 (TF and NFκB) or *n =* 6 (TNF-α) experiments each performed in duplicate. * Different to the control, *p <* 0.05; # Different to cells treated with SAA alone *p* < 0.05.

The effects of SAA have been postulated to be initiated by its binding to specific cell-surface receptors, including formyl-peptide receptor-like 1 (FPRL-1, also known as FPR2), Toll-like receptors 2 and 4 (TLR2/4) and Receptor for Advanced Glycation Endproduct (RAGE) [32]. Pharmacological inhibitors were employed targeting these receptors in an attempt to suppress SAA activity in vascular endothelial cells. Thus, cultured HCtAEC’s were pre-incubated with esRAGE, OxPapC (inhibitor of TLR2/4) or WRW4 (antagonist for FPRL-1) before SAA treatment and the mRNA levels of TF, TNF and NFκB were compared to those found with SAA treatment in the absence of added inhibitor (exemplar gel shown in Figure 1, and data summarised in Table 1). Pre-incubation of cells with the TLR2/4 inhibitor, OxPapC, significantly reduced SAA-induced elevated levels of all tested pro-atherogenic genes, TF, TNF and NFκB (Table 1). A higher dose of OxPapC (~2-fold) was also assessed however no increased modulation in gene regulation was noted when compared to the lower dose.

The FPRL-1 receptor antagonist, WRW4, significantly decreased SAA-induction of TNF and NFκB mRNA, but had no significant effect on TF mRNA levels (Table 1). In contrast, pre-treatment with esRAGE significantly decreased SAA-induced elevated TF mRNA but was less effective in inhibiting TNF and NFκB mRNA (Figure 1 and Table 1). Adding WRW4 to OxPapC in either dose produced no significant difference from cells pre-treated with OxPapC or WRW4 alone in inhibiting SAA modulation of TF or NFκB, though there was a non-significant trend to greater modulation of TF with the combination.

Next, we examined whether HDL confers protection from SAA-mediated pro-atherogenic effects in endothelial cells by pre-treating HCtAEC with 250 μg/mL (final concentration) of freshly isolated HDL. This dose of HDL corresponds to the lower quintile of HDL concentrations associated with cardiovascular disease in humans [40]. As shown in previous studies, HDL pre-treatment effectively reduced the elevated gene expression of TF, TNF and NFκB to near baseline levels determined for the control (no SAA) when compared to SAA-treatment alone (Table 1). Thus, pre-treatment with HDL reduced mRNA levels of TF, TNF and NFκB up to three times more than OxPapPC, WRW4 or esRAGE. The results indicate that pre-treatment of HCtAEC with HDL effectively mitigates SAA-induced pro-atherogenic gene expression (Table 1).

### 2.2. HDL Is a Chief Suppressor of SAA-Induced Pro-Atherogenic Protein Expression

**Figure 2:**
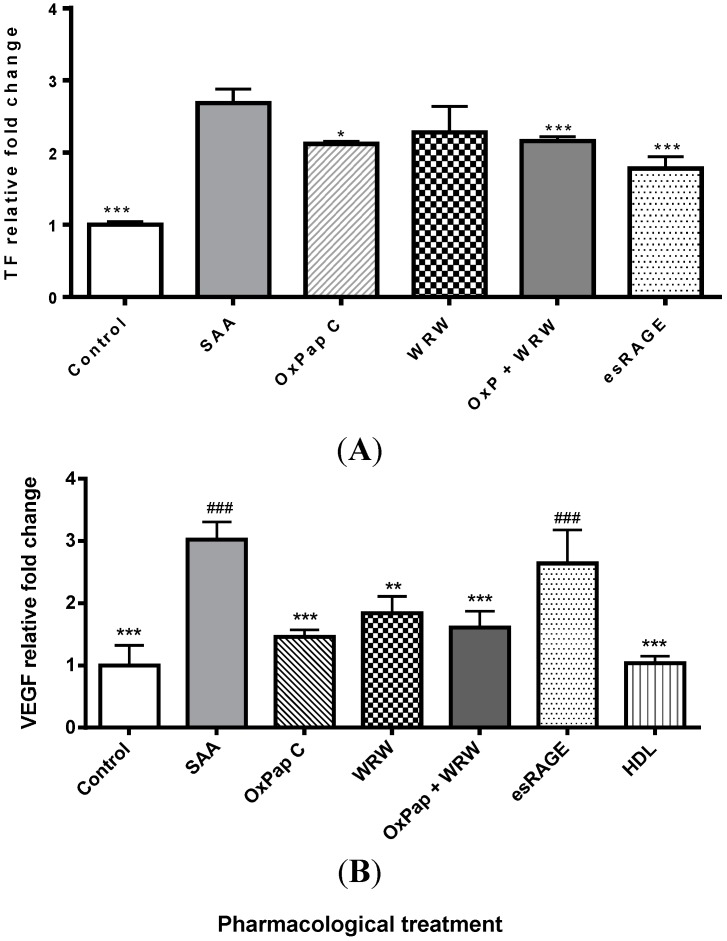
Suppression of SAA-induced TF and VEGF secretion by pharmacological agents and HDL. Cultured HCtAEC were treated with either vehicle (control) or pre-incubated with the indicated inhibitor (WRW4, 30 µg/mL; esRAGE, 15 µg/mL and OxPap C, 25 or 45 µg/mL) prior to the addition of SAA (10 μg /mL). Cells were incubated for 4.5 h at 37 °C, harvested and the level of (**A**) TF and (**B**) VEGF protein assessed by ELISA. Data represents mean ± SD of *n =* 3 independent experiments, each performed in triplicate and expressed as fold-change relative to control. * *p <* 0.05, ** *p <* 0.01 and *** *p <* 0.001 different from SAA treated group without pharmacological inhibitors. ^###^ *p <* 0.001 different from HDL pre-treatment group.

Treatment of cultured HCtAEC with SAA significantly increased secretion of TF (*p <* 0.001) (Figure 2A) and VEGF proteins (Figure 2B) (*p <* 0.001), the latter being a downstream response to NFκB activation via TNF [39]. The inhibitors, OxPapC and esRAGE, as well as native HDL were able to significantly inhibit the secretion of TF (*p <* 0.001) following SAA treatment (Figure 2A). WRW4 pre-treatment alone showed a non-significant decrease in TF secretion following SAA treatment, (*p =* 0.2). There was no further decrease in TF secretion with combined OxPapC and WRW4 pre-treatment compared to OxPapC or WRW4 alone. Native HDL pre-treatment significantly decreased SAA-induced VEGF secretion (*p <* 0.001) more effectively than any of the pharmacological inhibitors tested (Figure 2B).

SAA receptor inhibitors OxPapC and WRW4, and also HDL significantly reduced SAA stimulated secretion of VEGF however esRAGE pre-treatment had no effect (Figure 2B). Notably, HDL pre-treatment was significantly more effective in reducing SAA-induced VEGF secretion than esRAGE pre-treatment, whereas it was only slightly, but not significantly, more effective than WRW4 (*p =* 0.07) or the combination of OxPapC and WRW4 (*p =* 0.3).

**Figure 3:**
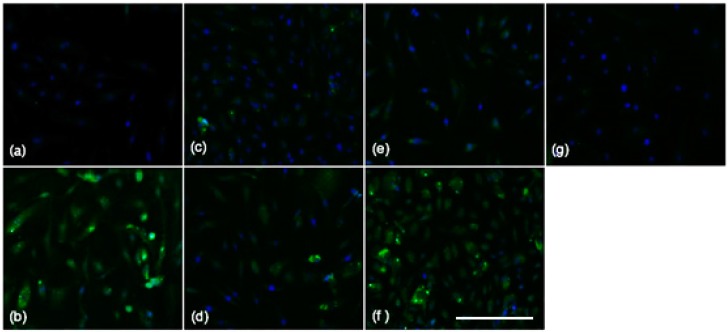
HDL suppresses SAA-induced VEGF secretion. Cultured HCtAEC were treated with vehicle (control) or pre-incubated with the indicated inhibitor (WRW4, 30 µg/mL; esRAGE, 15 µg/mL and OxPap C, 25 or 45 µg/mL) prior to the addition of SAA (10 μg/mL). Next, the cells were washed, fixed, permeablised and incubated with rabbit anti-VEGF mAb (1:200 *v*/*v*). Positive labelling was confirmed with a FITC conjugated anti-goat IgG antibody imaged at 40× magnification. (**a**) Represents control cells, or cells exposed to SAA in the presence of (**b**) no inhibitor; (**c**) OxPap C; (**d**) WRW4; (**e**) OxPap + WRW4; (**f**) esRAGE and (**g**) HDL. Scale bar = 150 µm.

The modulation of SAA-induced VEGF secretion by receptor inhibitors and HDL was examined further by assessing VEGF protein expression in HCtAEC by immunocytochemistry (Figure 3). Immune-fluorescent labelling of VEGF revealed base-line positive staining of VEGF in the control HCtAEC group (Figure 3a). However, labelling of VEGF was intensely positive in SAA treated HCtAEC (Figure 3b). In comparison, cells pre-treated with the pharmacological inhibitors or native HDL showed relatively little VEGF labelling (Figure 3c–g). Specifically, OxPapC and WRW4 pre-treatment revealed a moderate reduction in VEGF labelling (Figure 3c,d), and the combination of OxPapC + WRW4 failed to decrease VEGF labelling further relative to OxPapC or WRW4 alone (Figure 3e). Addition of esRAGE resulted in little reduction in VEGF expression, suggesting that esRAGE does not efficiently block SAA-stimulation of VEGF protein expression (Figure 3f). Cells pre-treated with native HDL before exposure to SAA showed virtually no labelling of VEGF, indicating that HDL effectively blocks SAA-mediated VEGF expression on HCtAEC (Figure 3g).

### 2.3. Blocking SAA Activation of RAGE Largely Fails to Inhibit SAA Activities on HCtAEC

**Figure 4:**
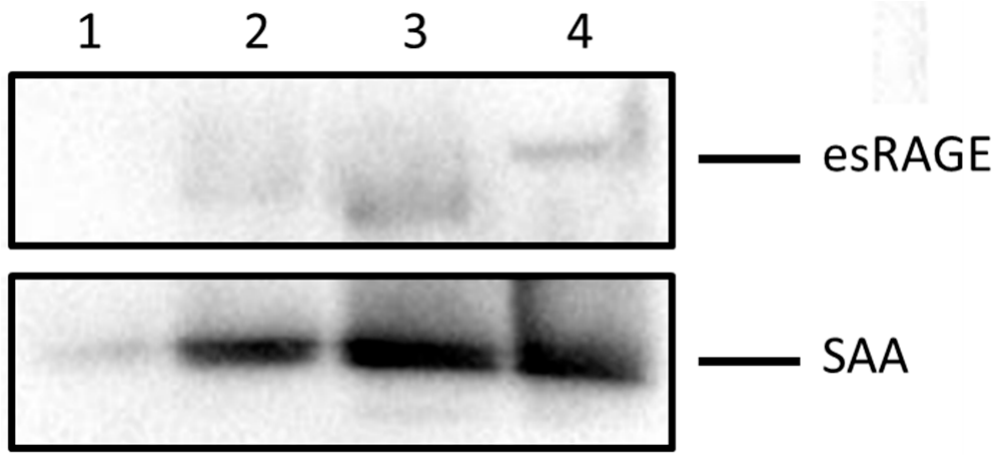
esRAGE partially binds to SAA as revealed by western blot analysis. Equal parts of esRAGE and recombinant SAA were mixed and incubated overnight at 4 °C. The mixture was immunoprecipitated using an anti-human esRAGE antibody conjugated to G-protein Sephorase beads. Immobilised proteins were then separated by 12% SDS-PAGE electrophoresis and subjected to immune-blotting using antibodies against esRAGE and SAA. Lanes represent: **1**-supernatant from wash number 3 of bead-protein complex; **2**-supernatant from wash number 2 of bead-protein complex; **3**-supernatant from wash number 1 of bead-protein complex; **4**-supernatant from unbound bead complexes after SDS and heat treatment. Data shows a single replicate representative of *n =* 3 independent studies with different SAA preparations.

Added esRAGE displayed a variable ability to modulate SAA-induced pro-atherogenic gene and protein expression in HCtAEC (Figure 2 and Table 1). Co-incubation of SAA and esRAGE at a 1:1 mol/mol ratio, followed by co-immunoprecipitation and western blotting demonstrated that the esRAGE peptide effectively bound to SAA *in vitro* (Figure 4, lane 4) indicating that esRAGE can act as a decoy for SAA in the media bathing the cultured HCtAEC. Proteins separated from the supernatant taken after three washes of sepharose G coupled beads bound to the IgG anti-SAA complex are shown in Figure 4. The third wash in lane 1 showed little to no residual esRAGE, though some SAA was noted. Overall, little SAA and esRAGE were found in the supernatant before bead complex dissociation. The majority of SAA bound to esRAGE was monomeric (*M*_r_ ~13 kDa), indicating that it is not in the fibrillar form when used to stimulate RAGE.

### 2.4. SAA Displaces Apolipoprotein A-I (ApoA-I) in HDL in a Dose-Dependent Manner

**Figure 5:**
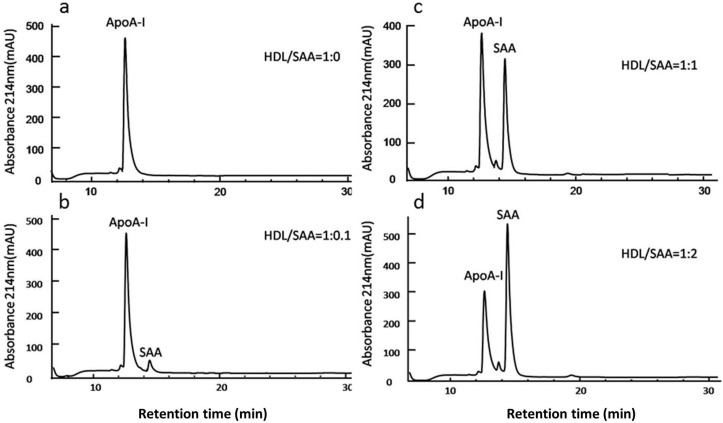
SAA displaces ApoA-I causing remodelling of the apolipoprotein profile of HDL. Freshly isolated HDL (250 μg/mL) was treated with vehicle (control) or SAA (at the final ratio indicated), and then dialysed against PBS to remove any free SAA. Proteins were then separated by reversed-phase chromatography using a linear acetonitrile gradient and monitoring at *A*_214 nm_. Chromatograms show altered HDL protein composition after exposure to SAA at ratios of HDL/SAA corresponding to: (**a**) 1:0; (**b**) 1:0.1; (**c**) 1:1 and (**d**) 1:2 mol/mol. Proteins (ApoA-I ~13 min) and SAA (~14.5 min) each eluted as a single symmetric peak.

Overall, HDL showed a superior efficacy in inhibiting SAA activities on HCtAEC, when compared to pharmacological inhibitors targeting FPRL-1, RAGE and TLR2/4. To demonstrate the SAA-sequestering ability of HDL, freshly isolated HDL was incubated with increasing molar ratios of SAA (0.1–2 SAA:HDL mol/mol) and levels of SAA and ApoA-I were assessed via HPLC to determine the apolipoprotein constituents of HDL. Under the experimental conditions employed, ApoA-I was the major protein peak identified in native HDL in the absence of SAA with a retention time of 13 min (Figure 5a). HDL incubated with 0.1 mol/mol SAA revealed an additional peak with a retention time of 14.3 min (corresponding to authentic SAA) with no detectable displacement of ApoA-I (Figure 5b). Increasing SAA to HDL ratios resulted in corresponding increases in the 14.3 min peak, correlating with SAA content (Figure 5c,d).

**Figure 6:**
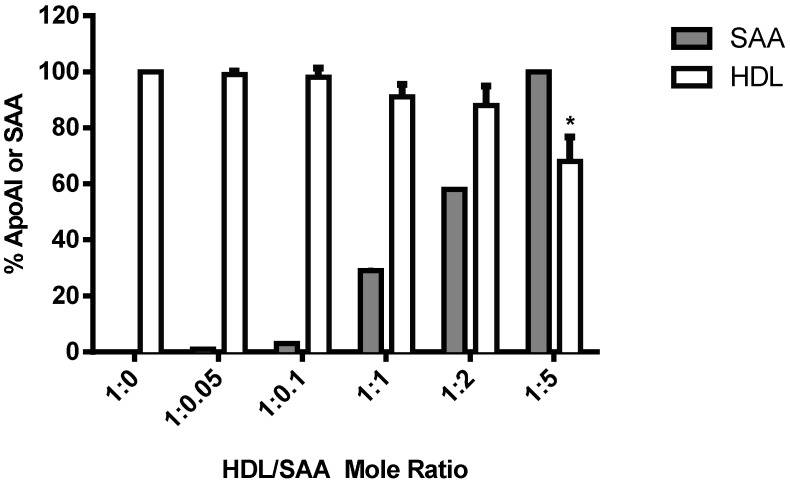
Changes in HDL apolipoprotein content in response to added SAA. The content of SAA (grey bar) and ApoA1 (white bar) in HDL were quantified using peak area analysis after separation with HPLC (see Figure 5). Changes in HDL apolipoprotein content were made after determining the maximal peak area response for ApoA-I and SAA and expressing peak areas as a percentage of that maximal area response (set to 100%). Data represent mean ± SD; *n =* 3 studies. * *p <* 0.05 compared to HDL in the absence of SAA.

Quantification of HDL content of both ApoA-I and SAA assayed by HPLC demonstrated an increasing concentration of bound SAA, and at a SAA/HDL ratio of 5 mol/mol, a significant decrease in the content of ApoA-I indicative of bound SAA stimulating the displacement of ApoA-I (Figure 6). Collectively the data indicates that SAA binds to HDL in a dose-dependent manner *in vitro* and can displace ApoA-I from native HDL when present at sufficiently high SAA/HDL ratios.

## 3. Discussion

Addition of SAA to HCtAEC significantly induced gene expression of the acute-phase nuclear transcription factor NFκB, the pro-inflammatory cytokine TNF and the pro-thrombotic protein TF and also elevated levels of the endothelial mitogen VEGF. The main novel outcome from our study was the differential modulation of SAA pro-atherogenic signalling in these cells by inhibitors/antagonists of the putative SAA receptors; FPRL-1, TLR2/4 and RAGE. Thus, OxPapC, a TLR2/4 receptor inhibitor, and the FPRL-1 antagonist, WRW4, inhibited NFκB, TNF and VEGF gene expression, while OxPapC (but not WRW4) modulated TF protein expression by added SAA. In contrast, the RAGE ligand decoy esRAGE blocked SAA mediated up-regulation of TF, but had little effect on any other pro-atherogenic activity mediated by SAA (NFκB, TNF or VEGF levels remained unaffected). A striking finding was that pharmacological blockade of SAA receptors was consistently less effective than adding freshly isolated human HDL. Thus, HDL readily incorporated SAA and markedly diminished SAA-induced pro-inflammatory and pro-thrombotic activity in HCtAEC. These data reinforce the notion that HDL effectively modulates SAA activity on the vascular endothelium and that circulating and/or tissue SAA:HDL ratios may be critical determinants of HDL’s protective activity. The findings also imply that there may be additional receptors for SAA, so complete blockade of SAA action may require a cocktail of inhibitors targeting multiple SAA receptors.

Previous studies have determined that exposure of endothelial cells to SAA stimulated pro-inflammatory/pro-thrombotic gene expression and a multi-angiokinase receptor inhibitor and HDL mitigated this gene response [24,39]. In this study, the ability of various known endothelial cell receptors for SAA were compared to HDL to determine efficacy against SAA-induced pro-atherogenic effects. With regard to specific receptor inhibition/antagonism, there appeared to be differential modulation of SAA-mediated cell signaling in cultured HCtAEC.

The TLR2/4 receptor inhibitor OxPapC, and the FPRL-1 antagonist, WRW4, showed a similar pattern of inhibition of SAA-induced pro-atherogenic genes/proteins, *i.e.*, effective against increased NFκB and TNF mRNA and VEGF protein expression, but less so or ineffective against TF mRNA/protein. Further, there appeared to be overlap or convergence of signaling pathways induced by SAA through these receptors, such that combination of both inhibitors produced no significant differences from either inhibitor alone. Cell signaling through TLR2/4 usually involves receptor dimerization and/or recruitment of co-receptors and NFκB and kinase activation are direct effects of SAA binding [39]. Similarly, NFκB and kinase activation occur with SAA interaction with FPRL-1 [41,42]. Our data further support NFκB activation as a common (pivotal step) in SAA pro-atherogenic signaling in endothelial cells via TLR2/4 and FPRL-1 receptors. Considering also that higher concentrations of OxPapC were no more effective in inhibition of responses than combination of OxPapC and WRW4, the data suggests regulation of residual NFκB, TNF or VEGF responses by SAA-receptors other than TLR2/4, FPRL-1 on these cultured EC.

NFκB induces the transcription of several pro-inflammatory and pro-thrombotic genes including TNF and VEGF and its activation is redox-sensitive. Both FPRL-1 and TLR2 are known to regulate intracellular calcium [43,44] that can impact the production of reactive oxygen species by mitochondrial and NADPH oxidase pathways in endothelial cells [24,25]. Interestingly, exposure of endothelial cells to peroxiredoxin-1 stimulates endothelial expression of VEGF through a mechanism involving TLR4 activation of hypoxia inducible factor that is sustained by NFκB activation in a feed-back loop [45], suggesting yet another link between the TLR/NFκB pathways and expression of VEGF. Unregulated oxidative stress activates redox sensitive NFκB leading to errant signalling and endothelial cell dysfunction [46].

TF up-regulation was only weakly inhibited by OxPapC, in contrast to TNF and VEGF, suggesting that TLR2/4 was not primarily involved in SAA-induced TF signaling in HCtAEC. Furthermore, a lack of inhibition by WRW4 indicated that FPRL-1 did not participate in TF responses although the opposite has been demonstrated for SAA in human umbilical vein endothelial (HUVEC) and human coronary artery endothelial cells [42]. In this latter study, SAA not only stimulated the expression of TF but also inhibited the expression of tissue factor pathway inhibitor through the activation of FPRL-1, leading to a stimulation of NFκB and MAP kinase-dependent pathways. These conflicting data may reflect differences in endothelial responses from different vascular beds to added SAA *i.e.*, umbilical or coronary endothelium compared to carotid endothelium (latter cell type used herein). The results are somewhat surprising given that TF is also driven by NFκB activation in various cell types [19]. However, inducible TF expression is transcriptionally regulated via binding of several diverse transcription factors to the TF promoter, including epidermal growth response-1 (Egr-1) and activator protein-1 (AP-1) factors and complex interactions may be required for TF gene expression or there may be functional redundancy of transcription activators.

esRAGE was moderately effective against SAA-induced TF mRNA/protein although it was ineffective in modulating SAA stimulated NFκB, TNF and VEGF responses. SAA binding to RAGE facilitates TF expression in monocytes [19] and RAGE signaling in endothelial cells activates NFκB and MAPK pathways leading to the activation of pro-inflammatory cascades possibly via oxidant-induced stress [47]. esRAGE modulation of SAA-induced TF was not accompanied by reduction in NFκB, again suggesting that NFκB is not primarily involved in SAA-induced TF expression in HCtAEC. Interestingly, esRAGE can interfere with RAGE signal transduction through two mechanisms including the formation of heterodimers with transmembrane RAGE at the cell surface [48] and sequestration of RAGE ligands through a decoy mechanism as we (Figure 4), and others [48] have demonstrated. Heterodimer formation exclusively blocks signalling through the RAGE receptor, whereas a decoy function for esRAGE can prevent the SAA ligand from activating other receptors such as TLR2/4 and FPRL-1. In our experimental model, cultured HCtAEC were preincubated with esRAGE prior to addition of SAA. This model likely favors direct blockade of RAGE through heterodimer formation although ligand binding may still contribute to the inhibitory activity determined here. In any case, ascertaining whether increasing the ratio of esRAGE to SAA might further mitigate SAA-stimulation of HCtAEC is warranted.

A recent report showed that SAA primarily up-regulated the expression of TLR2 in HCAEC, suggesting a positive feedback loop exists that amplifies SAA signalling [48]. Therefore, TLR2 may be the main functional receptor for SAA in endothelial cells, which could explain the overall high efficacy of gene inhibition by the TLR2/4 inhibitor, OxPapC in HCtAEC when compared to the FPRL-1 and RAGE antagonists. However, the data herein also show significant modulation of SAA pro-atherogenic activity via interaction with other SAA receptors in HCtAEC, suggesting the potential for complex SAA signalling interactions with various receptors on the endothelium.

In contrast to the pharmacological inhibitors/antagonists, HDL was able to almost completely abrogate SAA-induced pro-atherogenic activity in HCtAEC. Furthermore, only pre-treatment with HDL was able to reduce the expression of both TF and VEGF following SAA treatment. The results support previous data showing HDL dose-dependently inhibits SAA-stimulation of human aortic endothelial cells [22,24] and SAA-induced TNF-α release in THP-1 cells [49]. Thus, HDL appears to be an effective modulator of SAA activity on the endothelium. The anti-atherogenic effects of HDL are ascribed to its role in reverse cholesterol transport and its anti-inflammatory and anti-thrombotic functions on the endothelium [30].

Also, ApoA-1 appears to be necessary in mediating HDL’s protective effects on the endothelium. For example, ApoA-I enhances endothelial production of nitric oxide by interacting with endothelial nitric oxide synthase [50], which can result in improved vasomotor function as assessed in isolated vessels from apolipoprotein E-deficient mice administered ApoA-I [51]; actions that may potentially inhibit atherogenesis [52] and plaque rupture [53]. SAA is readily incorporated into HDL and the ability of SAA to displace ApoA-I at SAA/HDL ratios >5 mol/mol may yield lipid-poor ApoA-I, which has the potential to protect the endothelium and improve vascular function. However, in the studies employed here the SAA/HDL ratio was ~1:0.04 mol/mol (that is, SAA/HDL << 5), suggesting that SAA-mediated release of ApoA-I is unlikely to explain HDLs protective action on HCtAEC exposed to pro-inflammatory/pro-thrombotic SAA. Therefore, the HDL particle alone or HDL containing SAA may mediate anti-atherogenic effects on HCtAEC.

While HDL may simply reduce the bioavailability of SAA for cell signaling, thereby modulating SAA pro-atherogenic activities in endothelial cells, other mechanisms are, however, also likely. For example, it is well documented that HDL promotes cholesterol efflux via the ATP-binding cassettes ABCA1 and ABCG1 that modulate the fluidity of the plasma membrane as well as lipid raft formation [54]. Fluidity of the plasma membrane, specifically in microdomains, regulates the expression and distribution of membrane receptors. For example, it was recently demonstrated that deficiency of ABCA1 and ABCG1 on macrophages up-regulates the expression of TLR4 [55] and TLR2 [56,57] in the plasma membrane. Also, circulating HDL may indirectly inactivate or limit the activity of membrane receptors via a general modulation of membrane cholesterol levels [58]. Further investigations are warranted to determine whether cholesterol efflux via HDL changes the distribution or activity of SAA receptors such as RAGE and FPRL-1.

Whether SAA incorporated in HDL is pro- or anti-atherogenic is the subject of recent discordant findings. For example, in the bloodstream, SAA binding to vascular proteoglycans can result in the retention of pro-atherogenic lipoproteins to the vulnerable endothelium [33]. However, SAA-bound to HDL has been shown to enhance cholesterol efflux from lipid-loaded macrophages, compared to native HDL [59] suggesting an anti-atherogenic action for SAA. Also, there is data to indicate that while SAA facilitates HDL binding to cholesterol-loaded macrophages [60] it can subsequently impair the ability of HDL to promote cholesterol efflux, which may be related to displacement of Apo A-I [61]. Furthermore, a recent study failed to identify a link between SAA and atherosclerosis [62]. Similarly, apolipoprotein E-deficient mice deficient in endogenous SAA show no differences in aortic lipid deposition when fed either normal chow or a lipid-rich Western diet [63], although SAA may act to accelerate atherosclerosis rather than act as a causal agent [20]. Despite these disparate findings, the results presented herein show a clear anti-atherogenic effect of HDL on SAA activity on endothelial cells.

In summary, we have shown that SAA receptors, RAGE, TLR2/4 and FPRL-1 differentially modulate pro-atherogenic activity in endothelial cells and that single and dual inhibition of SAA receptors only partially abrogated SAA-mediated effects. Notably, HDL consistently conferred the highest efficacy of protection, likely through sequestration of SAA and thereby systemically curtailing interaction of SAA with receptors including, RAGE, TLR2/4 and FPRL-1. Therefore, individuals with normal (or high) circulating HDL levels may be inherently protected from SAA-mediated endothelial dysfunction, where HDL may act as a sink for SAA.

## 4. Methods

### 4.1. Materials

The following materials were obtained from Sigma–Aldrich (Sydney, Australia); fetal bovine serum (FBS), phosphate buffered saline (PBS), bovine serum albumin (BSA) and 2× sodium dodecyl sulphate (SDS) Laemmli loading buffer (4% (*w*/*v*) SDS, 20% (*v*/*v*) glycerol, 10% (*v*/*v*) 2-mercaptoethanol, 0.004% (*w*/*v*) bromphenol blue, 0.125 M Tris HCl). All solvents for HPLC were from Merck-Millipore (Sydney, Australia). Total RNA was isolated using a commercial kit (Total mRNA Isolate II Mini Kit, Bioline (Sydney, Australia). Reagents for cDNA preparation and assessment of gene regulation using RT-PCR (Oligo (dT) 18 Primer mix (50 µM), RNase inhibitor (40 μg/mL), dNTP Mix (25 mM), Bioscript (200 μg/mL), 5× Reaction Buffer, 2× MyTaq Red) were also obtained from Bioline. The following antibodies were obtained from Abcam (Sapphire Biosciences, Sydney, Australia); polyclonal rabbit anti-human tissue factor (TF), polyclonal rabbit anti-human vascular endothelial growth factor (VEGF), fluorescein isothiocyanate (FITC) conjugated polyclonal goat anti-rabbit IgG. Antibodies obtained from Sigma (Sigma–Aldrich, Sydney, Australia) included: peroxidase conjugated -goat anti-rabbit IgG, -rabbit anti-mouse IgG and -rabbit anti-goat IgG, whereas biotinylated goat anti-rabbit IgG was obtained from Dako (Sydney, Australia) and a polyclonal rabbit anti-human antibody raised against the *N*-terminus of RAGE was from Merck Millipore (Sydney Australia). Recombinant SAA was obtained from PeproTech (Rocky Hill, NJ, USA) and is a consensus molecule of the SAA1 and 2 isoforms used in previous studies [24]. The pharmacological TLR2/4 inhibitor, OxPap C (InvivoGen, San Diego, CA, USA), and FPRL-1 antagonist peptide WRW4 (Sigma, Sydney, Australia), were obtained from commercial sources. The polyclonal anti-human SAA antibody was obtained as a kind gift from Professor Carolyn Geczy (University of New South Wales).

### 4.2. Preparation of the Soluble Antagonist Targeting RAGE (esRAGE)

A commercial codon-optimised cDNA sequence for endogenous secretory (es) RAGE was derived from the native sequence by GeneArt (Life Technologies, Grand Island, NY, USA), using a proprietary algorithm. The pMA plasmid containing this cDNA (5 µg) was suspended in ultrapure H_2_O (50 µL) and then transformed into SURE-2 competent *E. coli* (Stratagene, Santa Clara, CA, USA) grown on ampicillin-treated agar plates. Colonies were then selected for Mini-preparation and colony PCR assessment using Quick Mini-prep kit (Life Technologies, Carlsbad, CA, USA) to verify that plasmids contained the esRAGE construct. Once the inserts were verified, a colony was selected for expansion using a Quick Maxi-prep kit (Invitrogen, Carlsbad, CA, USA). Where required, plasmid DNA concentrations were estimated by measuring absorbance at 260 nm with a nanodrop 2000 spectrophotometer (ThermoScientific, Waltham, MA, USA). Restriction enzyme digestion using Notl and EcoRV (obtained from New England Biolabs, Maine, MA, USA) confirmed the presence of a band running at the expected size of 1113 base pairs after separation with agarose gel electrophoresis. The insert was amplified using esRAGE-specific primers and TOPO cloned into the mammalian expression vector pcDNA3.2 (Invitrogen, Mulgrave, Australia). The resultant pcDNA3.2 plasmids containing esRAGE cDNA were then transformed into One Shot Top-10 *E. coli*, subcultured and the plasmid reisolated using a Quick Mini-prep kit. Correct sequencing was confirmed once again with Mini-preparation, RT-PCR and electrophoresis. Expression of the esRAGE protein from this final construct was tested *in vitro* by transfecting the mammalian cell line HEK293D with the pcDNA3.2-esRAGE plasmid, collecting the supernatant after 48 and 72 h incubation and quantifying secreted esRAGE using a commercially available human esRAGE sandwich ELISA (*cat*# K1009-1, B-Bridge International, Cupertino, CA, USA).

### 4.3. Cell Culture

Commercial Human Carotid Artery Endothelial cells (HCtAEC) (Cell Applications, San Diego, CA, USA) were outgrown and cryopreserved at the third passage. HCtAEC were cultured in a complete medium comprising MesoEndo Cell Growth Medium (Cell Applications, San Diego, CA, USA) and supplemented with 10% (*v*/*v*) FBS, 2 mM l-glutamine, 100 units/mL penicillin and 15 μg/mL endothelial cell growth serum (ECGS, Millipore, Sydney, Australia). Cells were routinely maintained in 25 mL cell culture flasks at 37 °C in a humidified, 5% CO_2_ atmosphere (Nuaire, Plymouth, MA, USA). Studies were performed with cells grown to 80%–90% confluence yielding ~3 × 10^6^ cells/mL; maximum passage number 6 following outgrowth from the original vial.

### 4.4. Isolation of Native HDL

For experiments testing the anti-atherogenic effects of human HDL on SAA-activated HCtAEC, native HDL was isolated from human plasma as described previously [64]. Briefly, whole blood was freshly obtained using a 21G gauge syringe (Terumo, Tokyo, Japan) and dispensed into Heparinised Vacutainers (Becton Dickson, Sydney, Australia). Plasma was obtained by centrifugation at 86× *g* at 4 °C for 20 min (GPR Centrifuge, Beckman, Sydney, Australia). Potassium bromide (KBr; final density 3.816 g KBr/mL) was dissolved in plasma by gentle mixing [64]. Once dissolved, an 18G gauge blunt needle was used to underlay approximately 1.9 mL of density-adjusted plasma (*d* = 1.063 g/L) into a 5.1 mL quick seal tube (Beckman, Sydney, Australia) that was subsequently filled with ice cold 50 mM PBS (*p* = 1.006 g/mL), pH 7.4. Tubes were then capped, heat-sealed and placed in a TLA 100.4 rotor and centrifuged at 430,000× *g* at 15 °C for 3 h using a Optima™ TLX ultracentrifuge (Beckman, Sydney, Australia). The HDL layer was extracted with a 25G 1 mL syringe (Terumo, Tokyo, Japan) and stored at 4 °C. Prior to use, KBr was removed from HDL preparations using Sephadex G-25, NAP-10 columns (Pharmacia, Brisbane, Australia). Finally, the protein content of the purified HDL was determined using with the Bicinchonic acid protein assay (BCA) described below.

### 4.5. Measurement of Cellular Protein Content

Protein concentration was determined using the BCA assay (Sigma, Sydney, Australia). Colour development was measured at *A*_562 nm_ against the standard bovine serum albumin (fraction V) (Sigma, Sydney, Australia) using a FLUOStar Omega Microplate Reader (BMG Labtech, Mornington, Australia).

### 4.6. Treatment of HCtAEC with SAA, HDL and Specific Receptor Inhibitors

Cultured HCtAECs were grown to 80%–90% confluence in 6-well plates and washed with Hanks’ Balanced Salt Solution (HBSS). HCtAECs were then overlaid with HEPES-buffered physiological salt solution (HPSS, pH 7.4), and either treated with vehicle (control) or with SAA (10 µg/mL) and incubated for 4.5 h at 37 °C under 5% CO_2_. As SAA activation of endothelial cells may occur via multiple receptors [48], different receptor antagonists were combined in an attempt to interrupt and dampen pro-atherogenic effects. Pharmacological antagonists to membranous FPRL-1, RAGE and TLR 2/4 were added to the cells prior to treatment with SAA. The respective inhibitors-WRW4 (final concentration 30 µg/mL), esRAGE (15 µg/mL) and OxPap C (25 or 45 µg/mL) were preincubated for 1.5 h at 37 °C prior to the addition of SAA (10 µg/mL). In other experiments, HCtAECs were pre-incubated with 250 µg/mL of freshly isolated HDL at 37 °C for 45 min prior to the addition of SAA (10 µg/mL). This relatively low HDL concentration is linked to enhanced cardiovascular disease in humans [40]. Independent experiments (*n =* 3) were performed in triplicate for all groups. Cell medium was then removed and the cell pellet isolated by centrifugation and designated to total RNA extraction or biochemical analysis.

### 4.7. Synthesis of Cloned DNA

Total RNA was isolated from cell samples using an Isolate II RNA Mini Kit as described in the manufacturer’s instructions (Bioline, Sydney, Australia). The quality and total concentration of the eluted RNA was determined by a ND-1000 UV–Vis Spectrophotometer (NanoDrop, Sydney, Australia). Next, cDNA was synthesised as described previously [65], using BioScript reverse transcriptase and Oligo-dT priming (Bioline, Sydney, Australia). Briefly, reactions were prepared by mixing the following reagents: 2 µL of isolated mRNA, 1 µL Oligo (dT) 18 µL Primer mix (50 µM) and 9 µL of Milli-Q water. Samples were then denatured at 70 °C for 5 min and rapidly chilled to 4 °C before the addition of RNAse inhibitor (40 µg/µL), dNTP mix (25 mM), Reaction Buffer (5×), Bioscript (200 µg/µL) and Milli-Q water (total volume of 20 µL. Reaction mixtures were heated to 94 °C for 2 min to stop any further reaction. Multiple cDNA preparations were synthesised. Transcribed cDNA was stored at −80 °C until required for assessment of gene regulation.

### 4.8. Gene Analysis

Semi-quantitative RT-PCR was used to investigate the effects of SAA on selective gene expression in HCtAEC. Genes of interest included; tissue factor (TF), tumor necrosis factor (TNF) and nuclear factor-kappa B (NFκB). The ubiquitous protein β-Actin was used as an internal standard for normalising all gene expression. The primer sequences for β-Actin and the selected functional genes are listed in Table 2. Polymerase chain-reactions were carried out using an Eppendorf Mastercycler (Lomb Scientific, Sydney, Australia) and the products were separated using agarose gel electrophoresis. Where required gels were imaged using a G:Box Chemi HR16 bioimaging system (Syngene, Frederick, MD, USA) and quantified using ImageJ (freeware, *v*1.42) NIHS [66]. The relative expression of each target gene was normalised to the corresponding level of β-Actin and expressed as a fold-change relative to control samples.

**Table 2:** Gene specific primer sequences for RT-PCR ^a^.

cDNA	Forward (5' → 3')	Reverse (5' → 3')
β-Actin	GGACTTCGAGCAAGA	AGCACTGTGTTGGCG
TF	GTGACCTCACCGACGAGATT	CCGAGGTTTGTCTCCAGGTA
TNF	CAGAGGGCCTGTACCTCATC	GGAAGACCCCTCCCAGATAG
NFκB	CTGGAAGCACGAATGACAGA	TGAGGTCCATCTCCTTGGTC

^a^ Primers were synthesised by Pro-oligo (Sigma, Sydney, Australia) and were diluted to 10 µg/mL prior to use. Briefly, duplicate reactions containing 2–4 µL of template sample cDNA added to a Master Mix containing 1 µL of each of the respective forward and reverse primers set of the gene of interest and 12.5 µL of 2× MyTaq Red mix. Milli-Q water was then added, to a final volume of 25 µL.

### 4.9. Direct ELISA Quantification of Tissue Factor and Vascular Endothelial Growth Factor

Secreted TF and VEGF protein levels were assessed using media sampled from treated HCtAEC. Specific protein determinations were performed using a MaxiSorp Nunc MicroWell 96-well microplates (Thermo Fisher Scientific, Waltham, MA, USA) coated with 10 µL/mL of sample protein in Coating Buffer (0.1 M sodium carbonate, pH 9.6) to a final volume of 50 µL and incubated overnight at 4 °C. Excess buffer was removed and plate washed in ELISA Wash Buffer (0.05% (*v*/*v*) Tween-20 in PBS, pH 7.4). Plates were coated with 50 µL Blocking Buffer (1% (*w*/*v*) skim milk in PBS, pH 7.4) for 2 h at 20 °C.

Excess coating buffer was removed and replaced with polyclonal rabbit, anti-human TF (1:200 *v*/*v*) or polyclonal rabbit anti-human VEGF (1:200 *v*/*v*) and incubated further at 20 °C for 2 h. Plates were washed 3 times (5 min each) in ELISA Wash Buffer and incubated with biotinylated goat anti-rabbit IgG (1:5000 *v*/*v*) for 30 min at 20 °C. Plates were washed as described previously and incubated with avidin/HRP complex (1:5000 *v*/*v*, R&D Systems, Minneapolis, MS, USA) for 30 min at 20 °C. Finally, plates were washed 4 times in ELISA Wash Buffer (5 min each), treated with 1 mM ABTS (Merk Millipore, Sydney, Australia) solution and incubated at 20 °C in the dark to facilitate chromophore development. After 45 min, stop solution (1% (*w*/*v*) SDS in dH_2_O) was added to each well and the absorbance measured at 405 nm using a FLUOStar mega Microplate Reader (BMG Labtech, Mornington, Australia). All samples were assayed in duplicate and average values expressed as a fold-change relative to the vehicle-treated control.

### 4.10. Immunocytochemistry

HCtAEC were seeded (1 × 10^4^ cells/mL) and cultured to confluence on 100 mm (dia ∅) glass cover slips in 6-well plates. Cultured HCtAEC were washed with HBSS and treated with either HPSS alone (control) or pre-incubated with the respective pharmacological inhibitor or HDL as previously specified, prior to the addition of SAA (10 µg/mL). Cells were incubated at 37 °C for 4.5 h and the media removed. HCtAEC were then fixed using 4% *w*/*v* paraformaldehyde and incubated for 5 min at 20 °C. Cells were washed in PBS, pH 7.4 and permeabilised with 50 µL of Triton-X 100 (0.1% *v*/*v* in PBS) for 10 min at 20 °C. Coverslips were blocked with 10% *v/v* FBS in PBS (Blocking buffer) for 1 h at 20 °C and cells were subsequently labelled with polyclonal rabbit anti-human VEGF (final dilution 1:200 *v*/*v*) at 20 °C. After 1 h, the antibody solution was aspirated and cells were washed three times (5 min each) in PBS before incubation with polyclonal goat anti-rabbit IgG conjugated to FITC (1:100 *v*/*v*) for 1 h at 20 °C. Cells were washed in PBS (4 × 5 min each) and coverslips mounted onto slides using 10 µL of Slow fade Gold antifade reagent with 4',6-diamidino-2-phenylindole (DAPI) (Prolong, Cell Signalling, Sydney, Australia). Slides were imaged using an Olympus fluorescent microscope (Olympus Ltd., Notting Hill, Australia).

### 4.11. Protein Complex Immunoprecipitation (Co-IP)

Co-Immunoprecipitation (Co-IP) was used to investigate the functional domains of SAA responsible for receptor RAGE binding and subsequent endothelial dysfunction using the decoy target, esRAGE. Equal concentrations of SAA and esRAGE (1 µg/mL in complete media) were combined and incubated at 4 °C overnight with gentle rotary agitation. Immunolabelling was performed by adding 2 µg/mL polyclonal rabbit anti-human esRAGE antibody (final dilution 1:200 *v*/*v*) or IP Wash Buffer (containing 0.025 M Tris, 0.15 M NaCl, 0.1 mM EDTA, 1% (*v*/*v*) NP-40, 5% *v*/*v* glycerol, pH 7.4) alone (Control). The vials were then incubated for 2 h at 20 °C under rotary agitation. Protein G-Sepharose conjugated beads (GE™ Healthcare, Sydney, Australia, 100 µL) were added to each sample and mixed with rotary agitation for 1 h at 20 °C. Mixtures were then centrifuged at 2000× *g* for 1 min at 4 °C and the supernatant removed and discarded. The IP complexes were washed 3 times in IP Wash Buffer for 2 min by centrifugation and then resuspended in 2× Laemmli loading buffer (BioRad, Sydney, Australia). The complexes were boiled rapidly for 5 min at 95 °C to allow protein elution. Vials were centrifuged at 14,000× *g* for 1 min and the supernatant retained for immediate SDS-polyacrylamide gel electrophoresis.

### 4.12. SDS-PAGE and Western Blotting

Prior to protein separation, the total protein content of the samples was determined and 30 µg of each sample protein was loaded onto a 12% SDS-PAGE gel [24]. Equivalent loading between lanes was confirmed by staining of a second parallel gel (run under identical conditions) with Coomassie Brilliant Blue (Sigma, Sydney, Australia). In-gel proteins were electroblotted onto activated Polyvinylidene difluoride nitrocellulose membranes (PVDF) that were subsequently blocked with 1% *w*/*v* bovine albumin (Fraction V, Sigma, Sydney, Australia) for 2 h at 20 °C. The proteins esRAGE and SAA were then detected by incubation with a polyclonal rabbit anti-human esRAGE antibody (1:1000 *v*/*v*) and a rabbit anti-human SAA antibody (1:1000 *v*/*v*) overnight at 4 °C. Membranes were then washed in 0.1% (*v*/*v*) Tween-20 in PBS, pH 7.4 before incubation with secondary peroxidase conjugated goat anti-sheep IgG (final dilution 1:5000 *v*/*v*) for 1 h at 20 °C. Protein bands were visualised using Luminata Forte (Merk Millipore) and images were captured with a ChemiDocTM MP imaging system (Bio-Rad, Sydney, Australia). Files were exported as TIFF files and semi-quantified by densitometry using Image J (freeware *v*1.42) NIHS USA [66]. Where required, TIFF files were imported into PowerPoint (version 7) for final manipulation.

### 4.13. Analysis of HDL Apolipoproteins ApoAI and SAA with Liquid Chromatography

Recombinant SAA was mixed with freshly isolated HDL at varying ratios ranging from 0 to 5 mol/mol then dialysed (DispoDialyzer, molecular cut-off 30 kDa; Spectrum Laboratories, Rancho Dominguez, CA, USA) against 50 mM PBS to remove unbound SAA and/or displaced ApoA-I. Aliquots (50 μL) of the purified lipoprotein were removed and denatured with 150 μL of 8 M guanidine hydrochloride on ice, and subsequently analysed by high performance liquid chromatography (HPLC) using a 5-μm, 25 × 0.46 cm C18 protein and peptide column (Vydac, Hesperia, CA, USA) with a 300-Å pore size. Apolipoproteins were eluted with a gradient of Buffer A (0.1% *v*/*v* trifluoroacetic acid) and Buffer B (90% *v*/*v* acetonitrile/H_2_O) at 1 mL/min at 20 °C using an Agilent 1100 series pump (Santa Clara, CA, USA) and detected using UV absorbance (214 nm) as described previously [64] with the following modifications: The gradient was formed starting with 75% Buffer A and 25% buffer B and the content of acetonitrile was increased linearly to 55% over 25 min, then to 90% over a further 5 min. Eluting peaks were quantified using Standard Agilent software (ChemStation v B.03.01, Agilent Technologies, Sydney, Australia) by peak area comparison and expressed as a percentage of the maximal peak area for a given condition.

### 4.14. Statistics

Statistical analyses were performed using GraphPad Prism statistical software v5.0 (GraphPad, CA, USA). Data are expressed as the mean ± SD and differences determined using one-way ANOVA with Tukey’s post hoc test to compare mean values between all data groups. Significance was accepted at *p* < 0.05 and actual *p*-values are given in the respective figure legends.

## 5. Conclusions

The main outcome from this study is the finding that pharmacological blockade of SAA receptors inconsistently inhibited SAA activity and was overall markedly less effective than human HDL. Thus, HDL incorporated SAA and diminished SAA-induced pro-inflammatory and pro-thrombotic activity. Therefore, HDL is superior in modulating SAA activity on the vascular endothelium and the SAA:HDL ratio in circulating blood may be a critical determinant for the ability for HDL to protect the endothelium from SAA pro-inflammatory and pro-thrombotic activity.

## Abbreviations

SAA: Serum Amyloid A
EC: Endothelial cells
CVD: Cardiovascular disease
esRAGE: Endogenous secretory RAGE
TF: Tissue factor
HCtAEC: Human carotid artery endothelial cells
FPRL-1: Formyl peptide receptor-like-1
TLR2/4: Toll-like receptors-2/4
HDL: High-density lipoprotein
NO: Nitric oxide
TNF: Tumour necrosis factor
TLRs: Toll-like receptors
RAGE: Receptor for advanced glycation end products
ApoA-I: Apolipoprotein A-I
NFκB: Nuclear factor κ B
HUVEC: Human umbilical vein endothelial
Egr-1: Epidermal growth response-1
AP-1: Activator protein-1
VEGF: Vascular endothelial growth factor
HBSS: Hanks’ balanced salt solution
DAPI: 4',6-diamidino-2-phenylindole
Co-IP: Co-Immunoprecipitation
PVDF: Polyvinylidene difluoride nitrocellulose
FITC: Fluorescein isothiocyanate
